# 2202. Missed Opportunities for Penicillin Allergy De-labeling in Infectious Disease Clinics

**DOI:** 10.1093/ofid/ofad500.1824

**Published:** 2023-11-27

**Authors:** Meaghan Martinez-Palmer, Parisa F Khan, Teena Xu

**Affiliations:** Baylor College of Medicine, Houston, Texas; Michael E. DeBakey Veterans Affairs Medical Center, Houston, Texas; Baylor College of Medicine, Houston VA, Houston, Texas

## Abstract

**Background:**

90% of patients who report a penicillin allergy are not truly allergic and can safely receive penicillins. Patients labeled as “penicillin allergic” are more likely to receive non-beta lactam antibiotics and suffer worse clinical outcomes. In this study, we aimed to quantify the rate of missed opportunities for penicillin allergy de-labeling in patients seen in ID clinic.

**Methods:**

This single-center retrospective study identified patients with a documented penicillin allergy who attended an ID clinic appointment between 1/1/21 and 12/31/22. Allergy history was ascertained by chart review. Extracted data included date of reaction entry into the medical record, reaction type (IgE-mediated, unknown reaction, side effect, severe cutaneous adverse reactions/non-IgE-mediated), observed or historical reaction, severity of reaction, and documented tolerance of penicillin since initial reaction. Patients who had tolerated penicillins since reaction or had side effects were eligible for immediate de-labeling. Patients with unknown reactions, remote IgE-mediated allergies >10 years ago, or non-severe IgE-mediated allergies >5 years ago were considered candidates for further evaluation and penicillin allergy testing. All other reactions were considered ineligible for further evaluation.

**Results:**

Of the 83 patients reviewed, 54 had reactions suggestive of an IgE-mediated allergy (Figure 1). 99% of patients had historical, self-reported reactions. At the time of ID clinic visit, 31 patients were eligible for immediate de-labeling due to tolerance of penicillins since reaction. Only 4 patients were ineligible for further evaluation. Median duration of penicillin allergy label was 18.8 years. 12 patients were ultimately de-labeled by the time of chart review.

Figure 1

**Figure:**
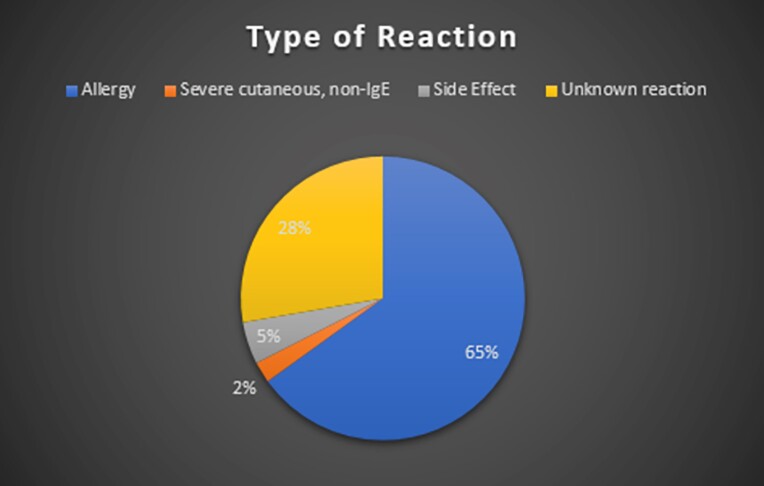

Types of Reactions

**Conclusion:**

37% of patients with penicillin allergies seen in an ID clinic were eligible for immediate allergy de-labeling. These findings demonstrate a high rate of missed opportunities for ID providers to remove penicillin allergies during clinic visits.

**Disclosures:**

**All Authors**: No reported disclosures

